# Feasibility of Flow Cytometric Bacterial Count in Synovial Fluid to Detect Periprosthetic Joint Infection in Hip and Knee Arthroplasty

**DOI:** 10.3390/jcm15103922

**Published:** 2026-05-19

**Authors:** Thomas J. A. van Schaik, Lex D. de Jong, Petra J. C. Heesterbeek, Saskia Susan, Jon H. M. Goosen, Job L. C. van Susante, H. W. Bart Schreuder, Janneke Ruinemans-Koerts

**Affiliations:** 1 Department of Orthopedic Surgery, Rijnstate Hospital, 6815 AD Arnhem, Gelderland, The Netherlands; 2 Department of Orthopedic Surgery, Radboudumc, 6525 GA Nijmegen, Gelderland, The Netherlands; 3 Department of Research, Sint Maartenskliniek, 6574 NA Ubbergen, Gelderland, The Netherlands; 4 Department of Orthopedic Surgery, Sint Maartenskliniek, 6574 NA Ubbergen, Gelderland, The Netherlands; 5 Department of Clinical Chemistry, Hematology and Immunology, Dicoon BV, Rijnstate Hospital, 6815 AD Arnhem, Gelderland, The Netherlands

**Keywords:** orthopedic surgery, periprosthetic joint infection, total hip arthroplasty, total knee arthroplasty, flow cytometry, bacterial count, synovial fluid

## Abstract

**Background**: Rapid and accurate preoperative diagnosis of periprosthetic joint infection (PJI) in patients undergoing revision arthroplasty remains challenging. Intraoperative differentiation between PJI and aseptic failure is limited by the lack of rapid and reliable diagnostic tools. Flow cytometric bacterial cell count of synovial fluid may represent a promising adjunct in this setting. This study explores the feasibility of using flow cytometric bacterial count of synovial fluid in the detection of PJI in patients during revision arthroplasty. **Methods**: In this exploratory pilot study, 93 patients provided informed consent. After exclusion of dry taps (*n* = 33), synovial fluid samples were collected intraoperatively from 60 patients undergoing revision total hip (THA) or total knee arthroplasty (TKA) at three hospitals in the Netherlands. Sixty samples were analyzed using the Sysmex UF-4000 to quantify flow cytometrically bacterial cells. Infection status was retrospectively determined according to the criteria of the European Bone and Joint Infection Society (EBJIS). Group differences were assessed using non-parametric analyses. **Results**: After exclusions due to dry taps and technical issues, samples of 43 patients could be analyzed. Of these patients, 16 had a confirmed PJI. Bacterial counts were significantly higher in patients with PJI compared with aseptic failure (median 1029 [IQR 287–4089] vs. 251 [IQR 92–433], *p* < 0.01). **Conclusions**: Flow cytometric synovial fluid analysis using the Sysmex UF-4000 demonstrated the ability to differentiate between patients with a PJI and with aseptic failure based on bacterial counts. However, this study was underpowered for diagnostic accuracy claims, and the feasibility of using flow cytometric bacterial count over established parameters such as total leukocyte and PMN% remains unclear at this stage.

## 1. Introduction

Total hip (THA) and knee arthroplasty (TKA) are among the most successful and commonly performed procedures in modern medicine, providing substantial pain relief and improving function and quality of life [1]. As procedure volumes continue to rise, the absolute number of complications is increasing, with revisions most often required for instability, aseptic loosening, periprosthetic fractures, and infection [2,3]. Periprosthetic joint infection (PJI) is one of the most devastating complications: with an incidence of 0.5–2%, it leads to prolonged morbidity, repeated surgeries, major healthcare costs, and an impact on survival and quality of life comparable to that of several common malignancies [4,5,6,7,8,9]. Diagnosing PJI remains a major challenge, as currently no diagnostic test offers absolute accuracy [10,11]. The Musculoskeletal Infection Society (MSIS) criteria [12] and the European Bone and Joint Infection Society (EBJIS) definition [13] have been considered the standard for diagnosis of PJI. These approaches integrate clinical assessment, peripheral blood and synovial fluid biomarkers, microbiological cultures, histological analysis, and intraoperative findings. Despite these advancements, preoperative diagnostic results are often inconclusive, and definitive confirmation typically depends on intraoperative tissue samples that require 10–14 days of culturing [14,15]. Ideally, infection status is determined intraoperatively as this would allow surgeons to promptly decide between septic or aseptic revision surgery and to initiate an appropriate antimicrobial regimen. Thus, there remains a need for a rapid and reliable intraoperative diagnostic tool.

Several intraoperative diagnostic techniques have been introduced to facilitate rapid identification of PJI [16,17]. These include synovial fluid-based assays such as the alpha-defensin lateral flow test (Synovasure^®^, CD Laboratories, Baltimore, MD, USA), synovial calprotectin measurement, intraoperative frozen section histology, and implant sonication followed by culture [17,18,19,20,21]. These methods have shown promising diagnostic performance, with reported high sensitivity and specificity in selected cohorts. However, despite these advantages, each technique has important limitations. Synovial biomarker tests may be affected by inflammatory comorbidities and can yield false-positive or false-negative results in certain clinical scenarios [16,20,22]. Consequently, while these methods are promising adjuncts, none have emerged as a definitive rapid intraoperative diagnostic standard.

Beyond conventional serum and synovial biomarkers such as C-reactive protein (CRP) and leukocyte count [20], flow cytometry has recently gained attention as a fast diagnostic approach for infectious diseases [23,24]. Flow cytometric synovial fluid analysis, including white blood cell (WBC) count and the percentage of polymorphonuclear cells (PMN%), is already widely implemented in the diagnostic work-up of PJI and demonstrates excellent diagnostic accuracy, with reported sensitivity and specificity often exceeding 95% [12,13,25,26,27]. These parameters form an essential component of current diagnostic criteria and are routinely used in clinical practice [12,13]. Nevertheless, situations such as periprosthetic fractures, inflammatory arthritis, crystal arthropathy, or adverse local tissue reactions due to metal debris may lead to elevated WBC counts or PMN% in the absence of infection, thereby reducing specificity [27]. These limitations highlight the need for complementary diagnostic approaches.

In contrast, flow cytometry can also provide an indication of bacterial presence, as automated systems such as the UF-4000 can detect bacterial counts in body fluids that correlate with Gram staining or culture positivity [24,28]. It is already implemented in the screening of urinary tract infections. However, the unique properties of synovial fluid, including its high viscosity, may limit assay performance, and this has not been systematically investigated. As an initial step, and with the long-term goal of finding a rapid intraoperative diagnostic test for PJI, the objective of this pilot study was to explore the feasibility of using flow cytometric bacterial counts in synovial fluid to detect differences between patients with PJI and those with aseptic failure undergoing revision THA or TKA.

## 2. Materials and Methods

### 2.1. Design and Participants

This exploratory pilot study recruited patients from three Dutch hospitals. Individuals of any age or sex scheduled for elective revision THA or TKA, involving the replacement of one or more fixed prosthetic components for septic or aseptic indications, were eligible for inclusion. Patients who were planned for revision of a mobile component only, administration of intravenous and/or oral antibiotics within two weeks prior to the diagnostic assessment or surgery, and refusal to provide consent for the use of their tissue in medical research were excluded.

### 2.2. Procedures

#### 2.2.1. Preoperative Procedures

**Figure 1:**
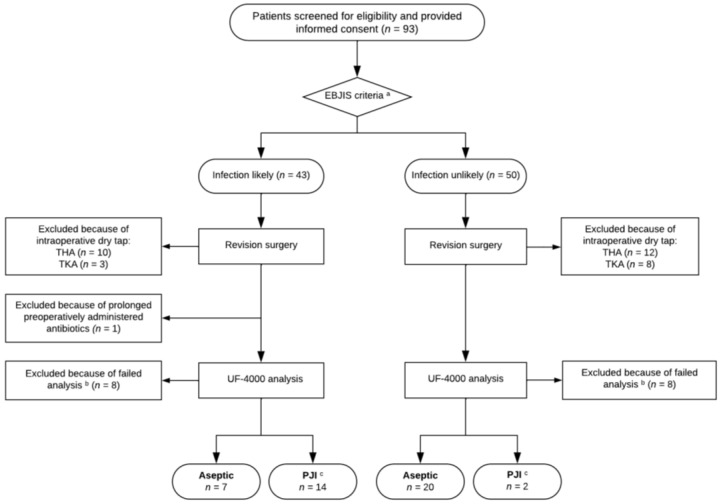
Flow diagram of inclusion procedure including study group allocation. ^a^ Patients allocated based on clinical features, including laboratory results and synovial fluid analysis; ^b^ samples excluded because of excessively viscous synovial fluid, blood admixture in the sample, or insufficient synovial fluid sample volume; ^c^ patients classified as PJI based on the EBJIS criteria of ‘Infection Confirmed’. EBJIS, European Bone and Joint Infection Society; THA, total hip arthroplasty; TKA, total knee arthroplasty; PJI, periprosthetic joint infection.

As part of the standard work-up prior to elective THA and TKA revision surgery, peripheral blood and synovial fluid samples were collected from all participants and analyzed. Blood samples were used to establish the patients’ serum levels of C-reactive protein (CRP). Synovial fluid was collected by sterile joint aspiration and analyzed for polymorphonuclear cells (PMN) cells and white blood cell count (WBC). These samples were obtained as part of the standard preoperative diagnostic work-up and were not collected specifically for the purpose of this study. Based on these preoperative analyses, patients were tentatively labelled ‘infection likely’ or ‘infection unlikely’ (see Figure 1) and subsequently received the type of surgery deemed appropriate by the surgeon.

#### 2.2.2. Intraoperative Procedures

All revision surgeries were performed according to a standardized protocol. The skin was disinfected using 0.5% chlorhexidine and patients were draped in a sterile fashion. Surgery was performed via a standard posterolateral approach with the patient in the lateral decubitus position. After dissection up to the posterior joint capsule and before performing the capsulotomy, the surgeon used a sterile needle and a 20 mL Luer-Lock syringe to collect one sample of at least 2.5–3.5 mL of synovial knee or hip joint fluid. In case of a dry tap, the participant was withdrawn from the study. The synovial fluid for analysis on the UF-4000 flow cytometer (Sysmex Corporation, Kobe, Japan) was kept in the syringe while being transported to the laboratory under refrigerated conditions. After joint aspiration and removal of the implant, and in line with standard practice, the surgeon collected six tissue samples of the interface membrane surrounding the hip or knee implant.

#### 2.2.3. Laboratory Analyses

Using the respective local hospitals available hemocytometry equipment, preoperative serum analyses were conducted, determining C-reactive protein (CRP). The intraoperatively collected synovial fluid was analyzed exclusively at the Department of Clinical Chemistry and Hematology of a single hospital within four hours of surgery using the UF-4000 flow cytometer (Sysmex Corporation, Kobe, Japan). No dilution or hyaluronidase treatment was performed; specimens considered excessively viscous based on visual assessment were excluded prior to measurement. Samples that could not be processed by the UF-4000 were excluded from further analysis. This device mixes the synovial fluid sample with fluorescent dyes to stain specific components of the cells (e.g., nucleic acids) before passing it through a laser beam in a flow cell. Sensors detected the light scatter and fluorescence signals of particles, allowing the system to count and classify particles based on size, shape, and internal structure. System algorithms provided counts of bacterial cells (BACT), mononuclear cells (MN) and their percentage (MN%), PMN and their percentage (PMN%), as well as red blood cells (RBC), total nucleated cells (TNC), and WBC.

### 2.3. Outcomes and Objectives

After all patients were preoperatively and tentatively classified as ‘infection likely’ or ‘infection unlikely’, their final infection status was retrospectively confirmed based on the six intraoperatively collected tissue specimens or other preoperative findings. The final diagnosis of PJI was based on any positive finding of the criteria for ‘Infection Confirmed’ as proposed by the EBJIS (4). Patients were subsequently analyzed within either the PJI or aseptic group. The main objective was to assess and compare flow cytometric bacterial counts between patients with PJI and those with aseptic failure.

### 2.4. Data Analysis

Participant characteristics were analyzed descriptively in terms of frequencies (percentages) and means (standard deviation). Infection status was analyzed descriptively in terms of frequencies (percentages). The medians (interquartile ranges) of the flow cytometry parameters were also analyzed descriptively, and between-group (PJI vs. aseptic, and infection likely vs. infection unlikely) differences were assessed using the Mann–Whitney U test.

All statistical analyses were performed using IBM SPSS Statistics (Version 30; IBM Corp., Armonk, NY, USA), with significance set at *p* ≤ 0.01.

## 3. Results

### 3.1. Patients’ Characteristics

Of the 93 patients who were screened for eligibility and provided informed consent, 33 were excluded at the time of revision surgery (Figure 1). In the group classified as ‘infection likely’, ten patients with THA and three with TKA were excluded due to an intraoperative dry tap. In the ‘infection unlikely’ group, twelve patients with THA and eight with TKA were excluded for the same reason. An additional 16 patients were excluded because the UF-4000 analysis failed due to excessively viscous synovial fluid, blood admixture in the sample, or an insufficient synovial fluid volume. Finally, one patient was excluded from the analyses because antibiotics had been administered more than two weeks prior to surgery. Characteristics of the 43 analyzable patients are summarized in Table 1. In brief, their mean age was 66.3 (10.1) years, and 19 were female. Based on revision surgery outcomes and microbiological culture results, 16 patients were classified as PJI and 27 as aseptic failure.

### 3.2. UF-4000 Parameters

**Figure 2:**
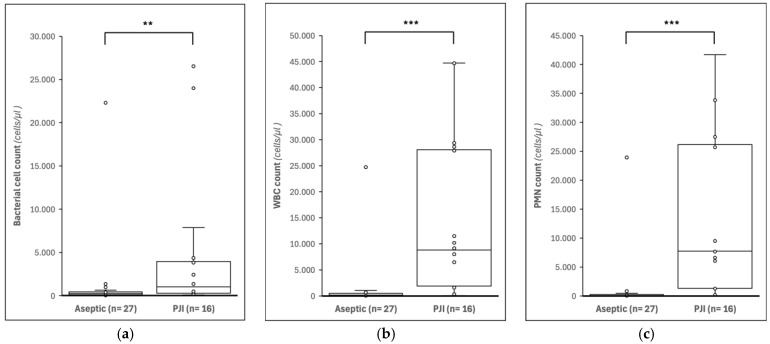
Boxplots of flow cytometric measurements by UF-4000 of synovial fluid in patients with aseptic failure and PJI. (**a**) Bacterial cell count, (**b**) white blood cell (WBC) count, and (**c**) polymorphonuclear (PMN) cell count are shown. ** indicates *p* < 0.01 and *** indicates *p* < 0.001 (Mann–Whitney U test).

Significant differences were observed in UF-4000 parameters between patients with PJI and with aseptic failure (Table 2). The bacterial cell count was higher in the PJI group compared to the aseptic group (*p* < 0.01). Inflammatory parameters, including WBC count, PMN count, and MN count, were also significantly higher and elevated in patients with PJI (all *p* < 0.001). TNC counts showed a similar pattern (*p* < 0.001). In contrast, RBC counts did not differ statistically between the groups (*p* = 0.766). Boxplots of bacterial cell count, WBC, and PMN (Figure 2) illustrate the higher values in the PJI group compared with aseptic samples (*p* < 0.01).

### 3.3. Outlier

One case within the aseptic failure group was identified as an outlier, showing markedly elevated bacterial cell count, WBC, and PMN values despite being classified as aseptic according to the EBJIS criteria (Table 3). This patient was classified as infection unlikely, as the preoperative diagnostic work-up revealed no abnormalities apart from radiological signs of loosening within the first five years after implantation. Additionally, intraoperative cultures showed no microbial growth. Flow cytometric analysis revealed markedly elevated bacterial cell count, WBC and PMN%.

## 4. Discussion

The results of this pilot study have demonstrated that several flow cytometry-derived parameters, including bacterial cell count, differed significantly between patients with PJI and aseptic failure. This suggests that flow cytometry of synovial fluids may have potential clinical utility in the diagnosis of PJI. However, the flow-cytometric approach was frequently limited by pre-analytical and technical issues (i.e., dry taps and sample viscosity), and these issues currently limit its diagnostic potential.

Automated synovial fluid analysis is increasingly recognized as a rapid and reliable diagnostic approach. Previous studies have demonstrated that synovial WBC count and PMN% provide high diagnostic accuracy, while biomarkers such as synovial CRP and calprotectin have also shown promising results [17,19,26,29,30,31]. Other intraoperative assays, including the alpha-defensin lateral flow test, have also been investigated to provide rapid detection of PJI. However, these tests can be influenced by inflammatory comorbidities and may yield false-positive or false-negative results in certain clinical scenarios. To our knowledge, flow cytometric quantification of bacterial cells in synovial fluid has not been previously evaluated in this context.

A clinically relevant advantage of the flow cytometry method is its speed and accessibility. The UF-4000 flow cytometer is already integrated into routine workflows for the analysis of urine and cerebrospinal fluid in clinical laboratories [24,28,32]. Applying this existing infrastructure to synovial fluid could reduce diagnostic turnaround time considerably compared to conventional microbiological culture, which typically requires 10–14 days. Faster intraoperative or early postoperative diagnosis of PJI could assist surgeons in faster decision-making between one-stage and two-stage revision procedures and in initiating appropriate antimicrobial therapy. The analysis can be performed immediately after synovial fluid collection, allowing results to become available early in the perioperative workflow, which is highly likely within approximately 30 min including transport and analysis. Consequently, flow cytometric analysis could potentially assist surgeons in considering the most appropriate revision strategy such as one-stage versus two-stage procedures during surgery.

### Limitations

Despite the potential advantages of using flow cytometry, several clinical utility issues came to light during this study. First, a relatively high rate in dry taps of 28% (24/84) was observed, both in the THA and TKA cases. While this rate is slightly lower than previously reported, it still limits the utility of flow cytometry in a substantial proportion of revision procedures [33,34,35,36]. In addition, during this study, aspiration was performed after surgical dissection up to the hip capsule. Preoperative percutaneous aspiration, as commonly performed in the clinical diagnostic setting, may be associated with an even higher risk of dry tap or contamination due to technical challenges and the lack of direct visualization.

In addition, we observed a high sampling error rate (28%; 17/60), most often due to high viscosity of the synovial fluid, insufficient sample volume, or blood contamination. While issues such as dry taps or blood admixture are primarily procedural and cannot be resolved by the analytical instrument itself, the high viscosity of synovial fluid may compromise flow cytometry performance. Enzymatic pretreatment, for example with hyaluronidase, could potentially reduce viscosity and improve assay reliability.

Combined, more than 50 percent of the initially included patients were excluded from the analyses, which has introduced partial verification bias or missing data bias, and limits the generalizability of our findings. This high exclusion rate should be considered when interpreting the data reported in this pilot study.

Although rapid analysis is deemed feasible, its integration into surgical workflows requires further investigation.

A noteworthy observation was the presence of an outlier in the aseptic failure group with elevated bacterial counts despite negative tissue cultures. This finding may reflect limitations of conventional culture techniques and raises the question of if flow cytometric bacterial detection could be used to identify bacterial presence in cases of culture-negative PJI [37,38,39]. This observation suggests there may be a clinically useful role for this technique in diagnostically challenging cases but warrants further investigation.

## 5. Conclusions

The results of this pilot study demonstrated that flow cytometric bacterial analysis of synovial fluid could differentiate between patients with PJI and patients with aseptic failure after revision arthroplasty. However, no robust conclusions could be drawn regarding its diagnostic value over, or as an adjunct to, established parameters such as synovial WBC count and PMN%, because the sample size was seriously limited due to a high exclusion rate of dry taps and substantial pre-analytical sampling issues. Future studies with larger sample sizes should focus on optimizing sample processing, improving analyzability, and evaluating the feasibility of rapid intraoperative implementation.

## Figures and Tables

**Table 1:** Patient characteristics.

	All	PJI *	Aseptic *
**Sample size,** n (%)	43	16 (37.2)	27 (62.8)
**Female**, n (%)	19 (44.2)	9 (30.0)	10 (48.5)
**Age**, mean ± SD	66.3 ± 10.1	66.6 ± 9.6	66.1 ± 10.5
**BMI**, mean ± SD	29.0 ± 5.0	28.5 ± 5.0	29.3 ± 5.0
**ASA-score**, n (%)			
ASA-1	-	-	-
ASA-2	34 (79.1)	14 (87.5)	20 (74.1)
ASA-3	7 (16.3)	1 (6.3)	6 (22.2)
ASA-4	2 (4.7)	1 (6.3)	1 (3.7)
**Joint**, n (%)			
Hip	13 (30.2)	5 (31.3)	8 (29.6)
Knee	30 (69.8)	11 (68.7)	19 (70.4)
**Type of revision surgery**, n (%)			
Tibial and femoral component revision	22 (51.2)	4 (25.0)	18 (66.7)
Explantation of prosthesis	10 (23.3)	9 (56.3)	1 (3.7)
Acetabular cup revision	4 (9.3)	-	4 (14.8)
Stem and acetabular cup revision	3 (7.0)	2 (12.5)	1 (3.7)
Stem revision	2 (4.7)	-	2 (7.4)
Conversion of UKA to TKA	2 (4.7)	1 (6.3)	1 (3.7)
**Preoperative analysis,** mean ± SD			
Serological CRP (mg/L)	19 ± 34	41 ± 47	6 ± 9
Synovial leukocyte count (10^9^/L)	11.0 ± 23.8	27.1 ± 32.9	0.7 ± 0.7
Synovial PMN (%)	55 ± 34	87 ± 9	28 ± 19
Preoperative suspicion of PJI *, n (%)			
Infection likely	21 (48.8)	14 (87.5)	7 (25.9)
Infection unlikely	22 (51.2)	2 (12.5)	20 (74.1)

* Based on the EBJIS definition for PJI. ASA, American Society of Anesthesiologists; BMI, body mass index; CRP, C-reactive protein; EBJIS, European Bone and Joint Infection Society; PJI, periprosthetic joint infection; PMN, polymorphonuclear neutrophil; SD, standard deviation; TKA, total knee arthroplasty; UKA, unicompartimental knee arthroplasty.

**Table 2:** Comparison of UF-4000 parameters between PJI and aseptic failure.

Parameter	PJI (*n* = 16)		Aseptic (*n* = 27)		*p*-Value ^1^
BACT (cells/µL)	1029	(287–4089)	251	(92–433)	<0.01
WBC (cells/µL)	8829	(1780–28,266)	135	(47–492)	<0.001
MN (cells/µL)	625	(277–2105)	49	(15–229)	<0.001
MN (%)	16	(7–30)	46	(29–55)	<0.001
PMN (cells/µL)	7754	(1302–26,580)	72	(22–242)	<0.001
PMN (%)	85	(70–93)	54	(45–71)	<0.001
TNC (cells/µL)	8874	(1783–29,162)	143	(47–545)	<0.001
RBC (cells/µL)	5326	(874–12,134)	3944	(341–16,290)	0.766

All values are presented as medians (interquartile range, IQR). ^1^ Differences between the groups were assessed using the Mann–Whitney U test. PJI, periprosthetic joint infection. BACT, bacterial cell count; WBC, white blood cells; MN, mononuclear cells; MN%, percentage of MN; PMN, polymorphonuclear cells; PMN%, percentage of PMN; TNC, total nucleated cells; RBC, red blood cells.

**Table 3:** Pre- and postoperative characteristics of UF-4000 outlier based on the EBIJS definition of PJI.

**Study ID**	**11**
**Preoperative suspicion of PJI**	Infection unlikely
**Joint**	Hip
**Type of revision surgery**	Stem and acetabular cup revision
**Clinical features**	
(1) Radiological signs of loosening within the first five years after implantation	Yes
(2) Previous wound healing problems	No
(3) History of recent fever or bacteremia	No
(4) Sinus tract with evidence of communication to the joint or visualization of the prosthesis	No
**C-reactive protein** *(in mg/L)*	6
**Synovial fluid analysis**	
WBC (in 10^9^/L)	-
PMN (%)	-
Microbiology results	No growth
**Intraoperative tissue cultures**	No growth
**UF-4000 results**	
BACT (cells/µL)	22,308
WBC (cells/µL)	24,742
MN (cells/µL)	808
MN (%)	3
PMN (cells/µL)	23,933
PMN (%)	97
TNC (cells/µL)	27,695
RBC (cells/µL)	4421

EBJIS, European Bone and Joint Infection Society; PJI, periprosthetic joint infection; BACT, bacterial cell count; WBC, white blood cells; MN, mononuclear cells; MN%, percentage of MN; PMN, polymorphonuclear cells; PMN%, percentage of PMN; TNC, total nucleated cells; RBC, red blood cells.

## Data Availability

The original data presented in the study are openly available in FigShare at https://doi.org/10.6084/m9.figshare.31988475.

## Abbreviations

The following abbreviations are used in this manuscript:

ASA	American Society of Anesthesiologists
BACT	Bacterial cells
BMI	Body mass index
CRP	C-reactive protein
EBJIS	European Bone and Joint Infection Society
METC	Medical Research Ethics Committee
MN	Mononuclear cells
MN%	Percentage of mononuclear cells
MSIS	Musculoskeletal Infection Society
PMN	Polymorphonuclear cells
PMN%	Percentage of polymorphonuclear cells
PJI	Periprosthetic joint infection
RBC	Red blood cells
THA	Total hip arthroplasty
TKA	Total knee arthroplasty
TNC	Total nucleated cells
UF-4000	Sysmex UF-4000 flow cytometer
UKA	Unicompartimental knee arthroplasty
WBC	White blood cells

## References

[1] Learmonth I.D., Young C., Rorabeck C. (2007). The Operation of the Century: Total Hip Replacement. Lancet.

[2] Jones C.M., Acuña A.J., Jan K., Forlenza E.M., Della Valle C.J. (2025). Trends and Epidemiology in Revision Total Hip Arthroplasty: A Large Database Study. J. Arthroplast..

[3] Jones C.M., Acuna A.J., Forlenza E.M., Serino J., Della Valle C.J. (2025). Trends and Epidemiology in Revision Total Knee Arthroplasty: A Large Database Study. J. Arthroplast..

[4] Tande A.J., Patel R. (2014). Prosthetic Joint Infection. Clin. Microbiol. Rev..

[5] Kunutsor S.K., Whitehouse M.R., Blom A.W., Beswick A.D. (2016). Patient-Related Risk Factors for Periprosthetic Joint Infection after Total Joint Arthroplasty: A Systematic Review and Meta-Analysis. PLoS ONE.

[6] Ramos M.S., Benyamini B., Kompala V., Khan S.T., Kunze K.N., McLaughlin J.P., Visperas A., Piuzzi N.S. (2025). Periprosthetic Joint Infection Mortality After Total Hip Arthroplasty Is Comparable to 5-Year Rates of Common Cancers: A Meta-Analysis. J. Arthroplast..

[7] Campbell D.G., Davis J.S., De Steiger R.N., Lorimer M.F., Harries D., Harris I.A., Manning L., Lewis P.L. (2025). Long-Term Mortality Associated with Periprosthetic Infection in Total Hip Arthroplasty: A Registry Study of 4,651 Revisions for Infection. J. Bone Jt. Surg. Am..

[8] Kristensen N.K., Gundtoft P.H., Elmengaard B., Pedersen A.B., Lange J. (2026). Increased Mortality Following Periprosthetic Joint Infection After Total Hip Arthroplasty: A Microbiologically Verified Nationwide Cohort of 1,611 PJI Revisions. J. Arthroplast..

[9] Kurtz S.M., Lau E., Watson H., Schmier J.K., Parvizi J. (2012). Economic Burden of Periprosthetic Joint Infection in the United States. J. Arthroplast..

[10] Ahmed S.S., Haddad F.S. (2019). Prosthetic Joint Infection. Bone Jt. Res..

[11] Springer B.D., Cahue S., Etkin C.D., Lewallen D.G., McGrory B.J. (2017). Infection Burden in Total Hip and Knee Arthroplasties: An International Registry-Based Perspective. Arthroplast. Today.

[12] Parvizi J., Tan T.L., Goswami K., Higuera C., Della Valle C., Chen A.F., Shohat N. (2018). The 2018 Definition of Periprosthetic Hip and Knee Infection: An Evidence-Based and Validated Criteria. J. Arthroplast..

[13] McNally M., Sousa R., Wouthuyzen-Bakker M., Chen A.F., Soriano A., Vogely H.C., Clauss M., Higuera C.A., Trebše R. (2021). The EBJIS Definition of Periprosthetic Joint Infection. Bone Jt. J..

[14] Fernández-Sampedro M., Fariñas-Alvarez C., Garces-Zarzalejo C., Alonso-Aguirre M.A., Salas-Venero C., Martínez-Martínez L., Fariñas M.C. (2017). Accuracy of Different Diagnostic Tests for Early, Delayed and Late Prosthetic Joint Infection. BMC Infect. Dis..

[15] Van Schaik T.J.A., De Jong L.D., Van Meer M.P.A., Goosen J.H.M., Somford M.P. (2022). The Concordance between Preoperative Synovial Fluid Culture and Intraoperative Tissue Cultures in Periprosthetic Joint Infection: A Systematic Review. J. Bone Jt. Infect..

[16] Magruder M.L., Heckmann N.D., Lieberman J.R., Scuderi G.R., Lustig S., Parvizi J., Mont M.A. (2026). Novel Technologies in Periprosthetic Joint Infection: Emerging Diagnostics. J. Arthroplast..

[17] Carli A.V., Abdelbary H., Ahmadzai N., Cheng W., Shea B., Hutton B., Sniderman J., Philip Sanders B.S., Esmaeilisaraji L., Skidmore B. (2019). Diagnostic Accuracy of Serum, Synovial, and Tissue Testing for Chronic Periprosthetic Joint Infection After Hip and Knee Replacements: A Systematic Review. J. Bone Jt. Surg. Am..

[18] Deirmengian C., Madigan J., Kallur Mallikarjuna S., Conway J., Higuera C., Patel R. (2021). Validation of the Alpha Defensin Lateral Flow Test for Periprosthetic Joint Infection. J. Bone Jt. Surg. Am..

[19] Stone W.Z., Gray C.F., Parvataneni H.K., Al-Rashid M., Vlasak R.G., Horodyski M.B., Prieto H.A. (2018). Clinical Evaluation of Synovial Alpha Defensin and Synovial C-Reactive Protein in the Diagnosis of Periprosthetic Joint Infection. J. Bone Jt. Surg. Am..

[20] Ruffier d’Epenoux L., Robert M., Lecomte R., Nich C., Bémer P., Corvec S., Chenouard R., Pailhories H., Tandé D., Lamoureux C. (2026). Synovial Biomarkers C-Reactive Protein and Calprotectin for Diagnosing Chronic Periprosthetic Joint Infection: A Prospective Multicenter Evaluation. J. Arthroplast..

[21] Sigmund I.K., Bue M., Kruse Jensen L., McNally M.A., Parvizi J., Sabater-Martos M. (2025). Histological Analysis in the Diagnosis of Periprosthetic Joint Infection of the Hip and Knee: A Systematic Review and Meta-Analysis. Bone Jt. J..

[22] Suen K., Keeka M., Ailabouni R., Tran P. (2018). Synovasure “quick Test” Is Not as Accurate as the Laboratory-Based α-Defensin Immunoassay: A Systematic Review and Meta-Analysis. Bone Jt. J..

[23] Oyaert M., Delanghe J. (2019). Progress in Automated Urinalysis. Ann. Lab. Med..

[24] Rubio E., Zboromyrska Y., Bosch J., Fernandez-Pittol M.J., Fidalgo B.I., Fasanella A., Mons A., Román A., Casals-Pascual C., Vila J. (2019). Evaluation of Flow Cytometry for the Detection of Bacteria in Biological Fluids. PLoS ONE.

[25] Qu X., Zhai Z., Liu X., Li H., Wu C., Li Y., Li H., Zhu Z., Qin A., Dai K. (2014). Evaluation of White Cell Count and Differential in Synovial Fluid for Diagnosing Infections after Total Hip or Knee Arthroplasty. PLoS ONE.

[26] Higuera C.A., Zmistowski B., Malcom T., Barsoum W.K., Sporer S.M., Mommsen P., Kendoff D., Valle C.J.D., Parvizi J. (2017). Synovial Fluid Cell Count for Diagnosis of Chronic Periprosthetic Hip Infection. J. Bone Jt. Surg. Am..

[27] Sabater-Martos M., Clauss M., Ribau A., Sousa R., Wouthuyzen-Bakker M., Bauer T., Berbari E., Cortes-Penfield N., Dietz M., Esteban J. (2025). Differential Synovial Fluid White Blood Cell Count for the Diagnosis of Chronic Peri-Prosthetic Joint Infection—A Systematic Review and Meta-Analysis. J. Bone Jt. Infect..

[28] Dossou N., Gaubert I., Moriceau C., Cornet E., le Hello S., Malandain D. (2022). Evaluation of Flow Cytometry for Cell Count and Detection of Bacteria in Biological Fluids. Microbiol. Spectr..

[29] Keemu H., Vaura F., Maksimow A., Maksimow M., Jokela A., Hollmén M., Mäkelä K. (2021). Novel Biomarkers for Diagnosing Periprosthetic Joint Infection from Synovial Fluid and Serum. JBJS Open Access.

[30] Hantouly A.T., Salameh M., Toubasi A.A., Salman L.A., Alzobi O., Ahmed A.F., Hameed S., Zikria B., Ahmed G. (2022). Synovial Fluid Calprotectin in Diagnosing Periprosthetic Joint Infection: A Meta-Analysis. Int. Orthop..

[31] Trampuz A., Hanssen A.D., Osmon D.R., Mandrekar J., Steckelberg J.M., Patel R. (2004). Synovial Fluid Leukocyte Count and Differential for the Diagnosis of Prosthetic Knee Infection. Am. J. Med..

[32] Korsten K., de Gier A., Leenders A., Wever P.C., Kolwijck E. (2024). Using the Sysmex UF-4000 Urine Flow Cytometer for Rapid Diagnosis of Urinary Tract Infection in the Clinical Microbiological Laboratory. J. Clin. Lab. Anal..

[33] Squire M.W., Della Valle C.J., Parvizi J. (2011). Preoperative Diagnosis of Periprosthetic Joint Infection: Role of Aspiration. AJR Am. J. Roentgenol..

[34] Ong J., Tang A., Rozell J.C., Babb J.S., Schwarzkopf R., Lin D. (2022). Factors Predicting Hip Joint Aspiration Yield or “Dry Taps” in Patients with Total Hip Arthroplasty. J. Orthop. Surg. Res..

[35] Christensen T.H., Ong J., Lin D., Aggarwal V.K., Schwarzkopf R., Rozell J.C. (2022). How Does a “Dry Tap” Impact the Accuracy of Preoperative Aspiration Results in Predicting Chronic Periprosthetic Joint Infection?. J. Arthroplast..

[36] Treu E.A., Behrens N.F., Blackburn B.E., Cushman D.M., Archibeck M.J. (2024). A “Dry Tap” in Prosthetic Joint Infection Workup of Total Hip Arthroplasty Is Not Reassuring. J. Arthroplast..

[37] Wouthuyzen-Bakker M. (2023). Cultures in Periprosthetic Joint Infections, the Imperfect Gold Standard?. EFORT Open Rev..

[38] Lin L., Li J., Zhang C., Li J., Wu B., Huang Z., Lv J., Liu M., Li W., Zhang W. (2025). Comprehensive Analysis of Culture-Negative Periprosthetic Joint Infection with Metagenomic next-Generation Sequencing. Front. Cell. Infect. Microbiol..

[39] Tan T.L., Kheir M.M., Shohat N., Tan D.D., Kheir M., Chen C., Parvizi J. (2018). Culture-Negative Periprosthetic Joint Infection: An Update on What to Expect. JBJS Open Access.

